# Galectin-3 enhances monocyte-derived macrophage efferocytosis of apoptotic granulocytes in asthma

**DOI:** 10.1186/s12931-018-0967-9

**Published:** 2019-01-03

**Authors:** Melanie Erriah, Kavita Pabreja, Michael Fricker, Katherine J. Baines, Louise E. Donnelly, Johan Bylund, Anna Karlsson, Jodie L. Simpson

**Affiliations:** 1 Priority Research Centre for Healthy Lungs, The University of Newcastle, Newcastle, NSW Australia; 2 Airway Disease Section, National Heart and Lung Institute, Imperial College London, London, UK; 3 Department of Oral Microbiology and Immunology, Sahlgrenska Academy at the University of Gothenburg, Gothenburg, Sweden; 4 Department of Rheumatology and Inflammation Research, Sahlgrenska Academy at the University of Gothenburg, Gothenburg, Sweden

**Keywords:** Asthma, Galectin-3, Macrophage, Neutrophil, Efferocytosis, Inflammation

## Abstract

**Background:**

Galectin-3 is a 32 kDa protein secreted by macrophages involved in processes such as cell activation, chemotaxis and phagocytosis. Galectin-3 has previously been shown to improve the ability of airway macrophages to ingest apoptotic cells (efferocytosis) in chronic obstructive pulmonary disease (COPD) and may be of interest in non-eosinophilic asthma (NEA) which is also characterised by impaired efferocytosis. It was hypothesised that the addition of exogenous galectin-3 to monocyte-derived macrophages (MDMs) derived from donors with NEA would enhance their ability to engulf apoptotic granulocytes.

**Methods:**

Eligible non-smoking adults with asthma (*n* = 19), including 7 with NEA and healthy controls (*n* = 10) underwent a clinical assessment, venepuncture and sputum induction. MDMs were co-cultured with apoptotic granulocytes isolated from healthy donors with or without exogenous recombinant galectin-3 (50 μg/mL) and efferocytosis was assessed by flow cytometry. Galectin-3 expression and localisation in MDMs was visualised by immunofluorescence staining and fluorescence microscopy. Galectin-3, interleukin (IL)-6 and CXCL8 secretion were measured in cell culture supernatants by ELISA and cytometric bead array.

**Results:**

Baseline efferocytosis (mean (±standard deviation)) was lower in participants with asthma (33.2 (±17.7)%) compared with healthy controls (45.3 (±15.9)%; *p* = 0.081). Efferocytosis did not differ between the participants with eosinophilic asthma (EA) (31.4 (±19.2)%) and NEA (28.7 (±21.5)%; *p* = 0.748). Addition of galectin-3 significantly improved efferocytosis in asthma, particularly in NEA (37.8 (±18.1)%) compared with baseline (30.4 (±19.7)%; *p* = 0.012). Efferocytosis was not associated with any of the clinical outcomes but was negatively correlated with sputum macrophage numbers (Spearman *r* = − 0.671; *p* = 0.017). Galectin-3 was diffusely distributed in most MDMs but formed punctate structures in 5% of MDMs. MDM galectin-3 secretion was lower in asthma (9.99 (2.67, 15.48) ng/mL) compared with the healthy controls (20.72 (11.28, 27.89) ng/mL; *p* = 0.044) while IL-6 and CXCL8 levels were similar.

**Conclusions:**

Galectin-3 modulates macrophage function in asthma, indicating a potential role for galectin-3 to reverse impaired efferocytosis in NEA.

**Electronic supplementary material:**

The online version of this article (10.1186/s12931-018-0967-9) contains supplementary material, which is available to authorized users.

## Background

Asthma is a heterogeneous condition characterised by excessive inflammatory cell infiltration of the airways and airway hyperresponsiveness [1]. About 50% of people with asthma have normal proportions of sputum eosinophils, termed non-eosinophilic asthma (NEA) [2, 3]. This phenotype of patients has also been found to have a defect in airway macrophage (AM) efferocytosis, defined as the engulfment of apoptotic host cells by phagocytes [4]. Defective efferocytosis can lead to accumulation of dead cells in the airways, which can undergo secondary necrosis and degranulation, worsening the inflammation and symptoms [5–7]. Efforts to improve macrophage efferocytosis may provide a more targeted approach to regulate inflammation in NEA.

Galectin-3 is a 32 kDa β-galactoside binding lectin secreted by macrophages and is known to play a role in the regulation of efferocytosis [8–10]. In a mouse model of asthma, the absence of galectin-3 was associated with a decreased clearance of apoptotic cells and persistence of inflammation [8, 11]. In humans, galectin-3 levels have been reported to be lower in asthma, particularly in neutrophilic asthma compared with eosinophilic and paucigranulocytic asthma [12], suggesting that failed efferocytosis may be occurring as a result of a galectin-3 deficiency in the airways. Exogenous galectin-3 can enhance efferocytosis of apoptotic cells by monocyte-derived macrophages (MDMs) [11] as well as by AMs in patients with chronic obstructive pulmonary disease (COPD) [10] which highlights the potential of galectin-3 in the regulation of macrophage function in NEA.

This study characterised MDM efferocytosis in asthma and examined the effect of galectin-3 on this process. We hypothesised that MDMs from participants with asthma, particularly those with NEA would show reduced efferocytosis compared with healthy controls and that this defect would be reversed with the addition of exogenous galectin-3.

## Methods

### Participant population

Efferocytosis was assessed in blood MDMs derived from participants with stable asthma (*n* = 19) and healthy controls (*n* = 10). Participants with asthma had a confirmed doctor’s diagnosis of asthma with evidence of variable airflow obstruction such as airway hyper-responsiveness, bronchodilator response (improvement of 12% and 200 mL following a short acting bronchodilator) or diurnal variation of peak expiratory flow [13]. Participants who had experienced an asthma exacerbation or a change in asthma medications in the four weeks prior to the visit were ineligible for this study. The participants underwent a clinical assessment, which included the asthma control questionnaire 6 (ACQ6) [14], history of smoking, respiratory symptoms, skin prick allergy testing, sputum and blood collection. Ethics approval was granted by the Hunter New England Human Research Ethics Committee (Approval number: 15/12/16/4.01) and the University of Newcastle Ethics Committee (Approval number: H-2016-0114) and written informed consent was obtained.

Participants were excluded if their post-bronchodilator forced expiratory volume in one second (FEV_1_) was less than 40% predicted as a safety precaution or if they were currently smoking or had ceased smoking in the last twelve months to limit confounders. Ex-smokers with a smoking history of more than 10 pack years combined with an exhaled carbon monoxide > 10 ppm (ppm) were excluded.

### Sputum induction

Spirometry and sputum induction were performed as described previously [15]. Briefly, a fixed sputum induction time of 15 min with hypertonic saline (4.5%) was used for all participants. The sputum sample was then dispersed using dithiothreitol for a total cell and viability count [15]. May-Grunwald Giemsa-stained cytospin slides were also prepared and a differential cell count of 400 non-squamous cells was performed to identify the asthma sputum phenotype. Participants with ≥3% eosinophils were classified as eosinophilic asthma (EA) and those under 3% were considered as NEA [16].

### Generation of macrophages

Peripheral blood mononuclear cells (PBMCs) were isolated from whole blood by density gradient centrifugation using Percoll gradients and resuspended in RPMI (GE Healthcare, Parramatta, NSW, Australia) supplemented with 10% (v/v) foetal bovine serum (FBS; Bovogen Biologicals, Keilor East, VIC, Australia), 1% (v/v) streptomycin (Thermo Fisher Scientific, North Ryde, NSW, Australia), 2 mM L-glutamine (Thermo Fisher Scientific), 20 mM HEPES (GE Healthcare) at 4 × 10^6^ cells/mL. PBMCs were dispensed in a 24-well plate at 2 × 10^6^ cells/well and incubated for 2 h at 37 °C to allow the monocytes to adhere. The wells were washed three times with pre-warmed phosphate buffered saline (PBS) and the monocytes differentiated into MDMs over 12 days in culture media supplemented with 2 ng/mL granulocyte macrophage-colony stimulating factor (GM-CSF) (Sigma-Aldrich, Castle Hill, NSW, Australia) as described previously [17]. The media was replaced every 3 to 4 days. MDM culture supernatant was collected prior to the efferocytosis assay and stored at − 80 °C.

### Galectin-3 ELISA and immunocytochemistry

Galectin-3 levels were measured in MDM culture supernatant by ELISA (R&D Systems, Minneapolis, MS, USA) according to the manufacturer’s instructions. Galectin-3 expression and localisation in MDMs was also assessed by immunocytochemistry as described previously [18]. Following aspiration of the supernatant, 500 μL Accutase cell dissociation reagent (Thermo Fisher Scientific) was added to each well and incubated at 37 °C for 15 min to detach the MDMs. The cells were then transferred to cytospin slides, fixed in Paraformaldehyde/Lysine/Periodate (PLP) fixative (Sigma-Aldrich) as described previously [19] and coated in 15% (w*/*v) sucrose to protect them prior to storage at − 20 °C. The slides were thawed and the cells permeabilised with 0.1% Triton-X (Sigma-Aldrich) in phosphate buffered saline, blocked and immunostained with anti-galectin-3-Phycoerythrin (PE) (Biolegend, San Diego, California, USA) and primary anti-LAMP-1 antibody (US Developmental Studies Hybridoma Bank at the University of Iowa, Iowa City, USA) followed by secondary Alexa Fluor 488 anti-mouse antibody (Cell Signaling Technologies, Danvers, Massachusetts, USA). A PE-conjugated isotype control antibody (Biolegend) and an additional PE-conjugated galectin-3 antibody (BD Biosciences, North Ryde, NSW, Australia) were also included to rule out non-specific binding. The cells were mounted with ProLong Gold Antifade Mountant with 4′,6-diamidino-2-phenylindole (DAPI) (Thermo Fisher Scientific). Slides were observed on an Axio Imager.M2 epifluorescence microscope (ZEISS, Macquarie Park, NSW, Australia) under fluorescent optics and images were taken using an Axiocam 506 mono camera (ZEISS).

### Cytometric bead array

The concentration of IL-1β, IL-6, IL-8, IL-10, IL-17A, IL-17F, IFN-ɣ and TNF was measured in MDM supernatant using a CBA Flex Set from BD Biosciences. Standard curves were reconstituted according to the manufacturer’s instructions. The samples were used neat and the assay was performed in Titertube Micro Test Tubes (Bio-Rad, Gladesville, NSW, Australia). All incubation steps were performed at room temperature and away from light. Capture beads (50 μL) were added to each titertube and incubated for one hour. PE detection reagent (50 μL) was then added to each titertube, mixed and incubated for 2 h. The beads were washed and resuspended in 300 μL of Wash Buffer. The tubes were vortexed briefly and loaded on a 96-well plate (Greiner Bio One, Kremsmünster, Austria) using a Viaflo II multichannel electronic pipette (Integra Biosciences, Zizers, Switzerland). The 96-well plate was then loaded in a FACS Canto II flow cytometer and a total of 300 events per target was recorded per well. The data was analysed using the FCAP array software version 3.0 (BD Biosciences).

### Granulocyte labelling

Granulocytes were isolated from whole blood of healthy volunteers by density gradient centrifugation using Percoll and labelled with 2.5 μM PKH26 (Sigma-Aldrich) as described previously [20]. The labelling reaction was stopped after 5 min by the addition of FBS and the cells washed twice with PBS. The granulocytes were resuspended at 1 × 10^6^ cells/mL in RPMI supplemented with 1% (v/v) FBS and 1% (v/v) penicillin/streptomycin and cultured for at least 20 h to induce apoptosis. This was found to result in > 80% apoptotic cells measured using the FITC Annexin V apoptosis detection kit from BD Biosciences.

### Efferocytosis assay

Adherent MDMs were pre-incubated with 50 μg/mL galectin-3 (kindly donated by Professor Anna Karlsson, University of Gothenburg, Sweden) (or media) in complete media for 10 min at 37 °C prior to efferocytosis [11]. Apoptotic granulocytes were co-cultured with MDMs at a ratio of 5:1 for 90 min at 37 °C [20]. The MDMs were then washed thrice with 500 μL PBS to remove unbound granulocytes and detached using Accutase as described earlier. The cells were transferred to FACS tubes and efferocytosis was measured by flow cytometry (FACS Canto II, BD Biosciences). A total of 10,000 macrophages were recorded based on forward and side scatter characteristics and the median fluorescence intensity (MFI) was also recorded. The efferocytosis assay was also performed in the presence of 5 μg/mL cytochalasin D (including a 30 min pre-incubation step) to control for surface-bound but non-internalised granulocytes [21]. Data was analysed using FACSDiva version 8 and net efferocytosis was reported as the difference in the percentage of PKH26-positive MDMs in the presence and absence of cytochalasin D.

### Statistics

All results are expressed as mean ± SD or median (q1, q3) unless otherwise indicated. The unpaired t test (parametric data) and Mann-Whitney U test (non-parametric data) were used for comparisons between unpaired samples and the paired t test used for paired data. The two-sample t-test of proportion was used to compare proportions and the Spearman rank correlation was used to identify associations between data. All statistical analyses were performed using GraphPad Prism software version 7.02 (GraphPad Inc., La Jolla, CA, USA) and Stata version 13.1 (Stata Corporation, College Station, TX, USA). A *p*-value < 0.05 was deemed statistically significant.

## Results

### Subject demographics

The participants with asthma were matched for age but had a higher BMI compared with the healthy controls (Table 1). The participants’ gender, smoking history and atopy did not differ between the asthma and healthy controls. The majority (79%; *n* = 15) of participants with asthma were taking ICS with a median daily dose of 1000 μg. The majority (68%; *n* = 13) of participants had moderate to severe asthma and were GINA step 4, while the rest were classified as GINA step 1 (21%; *n* = 4) and 3 (11%; *n* = 2).

**Table 1:** Participant clinical characteristics

	Asthma	Healthy controls	*p* value
*n*	19	10	
Age, years	50.0 (±18.8)	47.3 (±17.5)	0.707
Sex, male (%)	5 (26)	3 (30)	0.833
BMI	31.9 (±5.4)	25.1 (±3.7)	0.001
Atopy, *n* (%)	9 (75)	5 (56)	0.350
FEV_1_ predicted (%)	97.6 (77.8, 105.9)	108.7 (94.1, 113.7)	0.083
FEV_1_/FVC (%)	92.7 (±14.3)	102.3 (±5.4)	0.065
Ex-smokers, *n* (%)	4 (21)	2 (20)	0.947
Pack years	18.4 (±27.6)	7.5 (±8.8)	0.633
Exhaled CO (ppm)	3 (2, 4)	4 (2, 5)	0.825
Taking ICS, *n* (%)	15 (79)	N/A	N/A
ICS dose BDP equivalent	1000 (800, 1000)	N/A	N/A
GINA treatment step, *n* (%):			
1	4 (21)	N/A	N/A
3	2 (11)	N/A	N/A
4	13 (68)	N/A	N/A

Data is reported as mean ± SD or median (q1, q3). Data was analysed by the two sample t-test or Mann-Whitney U test. The two-sample test of proportions was used to compare proportions. A p-value of < 0.05 was considered to be statistically significant. BDP: beclometasone dipropionate, where 1 μg of beclomethasone = 1 μg budesonide = 0.5 μg fluticasone; BMI: body mass index; FEV_1_: forced expiratory volume in one second; FVC: forced vital capacity; ICS: inhaled corticosteroid; GINA: global initiative for asthma; N/A: not applicable

The EA and NEA participants were of similar age, gender and BMI (Table 2). They were taking a similar dose of ICS and did not differ in their lung function.

The sputum total cell count and viability was similar between the EA and NEA phenotypes (Table 3). Participants with EA had the highest proportion and number of eosinophils as expected but similar macrophage, neutrophil and lymphocyte proportions.

**Table 2:** Clinical characteristics by asthma inflammatory phenotypes

	EA	NEA	*p* value
n	8	7	
Age, years	53.7 (±13.0)	49.7 (±22.0)	0.672
Sex, male (%)	1 (13)	2 (29)	0.438
BMI	33.7 (±4.7)	28.8 (±3.9)	0.057
Atopy,* n* (%)	5 (83)	3 (60)	0.387
Ex-smoker, *n*(%)	3 (38)	1 (14)	0.310
Pack years	4.8 (±6.3)	59.1	–
Exhaled CO (ppm)	4 (3, 7)	2 (2, 3)	0.051
FEV_1_ predicted (%)	84.9 (69.7, 102.3)	101.9 (82.5, 105.9)	0.189
FEV_1_/FVC (%)	92.0 (±16.1)	97.3 (±15.3)	0.522
Taking ICS, *n* (%)	7 (89)	6 (86)	0.919
ICS dose (μg/day)	1000 (800, 1000)	1000 (750, 1625)	0.422
ACQ6 score	1.3 (0.5, 2.3)	0.7 (0.2, 0.8)	0.112

Data is reported as mean ± SD or median (q1, q3). Data was analysed by the unpaired t test and Mann-Whitney U test. The two-sample test of proportions was used to compare proportions. ACQ6: asthma control questionnaire 6; BMI: body mass index; CO: carbon monoxide; FEV_1_: forced expiratory volume in one second; FVC: forced vital capacity; ICS: inhaled corticosteroid

**Table 3:** Sputum cell numbers by asthma inflammatory phenotypes

	EA	NEA	*p* value
*n*	8	7	
Total cell count (10^6^/mL)	5.4 (1.5, 10.1)	4.1 (3.7, 11.8)	0.965
Viability (%)	87.8 (64.6, 95.8)	70.5 (57.2, 94.3)	0.699
Neutrophils (%)	29.8 (23.8, 61.4)	50.3 (9.0, 55.0)	0.980
Neutrophils (10^4^/mL)	150.8 (38.5, 591.6)	211.8 (103.8, 700.9)	0.699
Eosinophils (%)	6.6 (6.1, 8.8)	1.0 (0.0, 1.5)	< 0.001
Eosinophils (10^4^/mL)	45.3 (9.1, 106.5)	4.6 (0.8, 6.9)	0.009
Macrophages (%)	39.1 (27.8, 63.4)	44.5 (35.0, 73.5)	0.536
Macrophages (10^4^/mL)	238.0 (94.0, 314.7)	251.4 (159.3, 459.8)	0.589
Lymphocytes (%)	5.1 (0.4, 6.4)	1.8 (1.0, 3.0)	0.381
Lymphocytes (10^4^/mL)	4.6 (1.2, 35.4)	9.4 (4.4, 20.2)	0.833
Columnar epithelial cells (%)	0.8 (0.1, 6.6)	2.8 (1.3, 10.0)	0.141
Columnar epithelial cells (10^4^/mL)	1.9 (0.0, 7.1)	14.0 (9.1, 28.9)	0.026
Squamous cells (%)	3.0 (0.7, 15.8)	6.3 (3.8, 19.5)	0.161

Data is reported as median (q1, q3). Data was analysed by the Mann-Whitney U test

### MDM efferocytosis

**Fig. 1:**
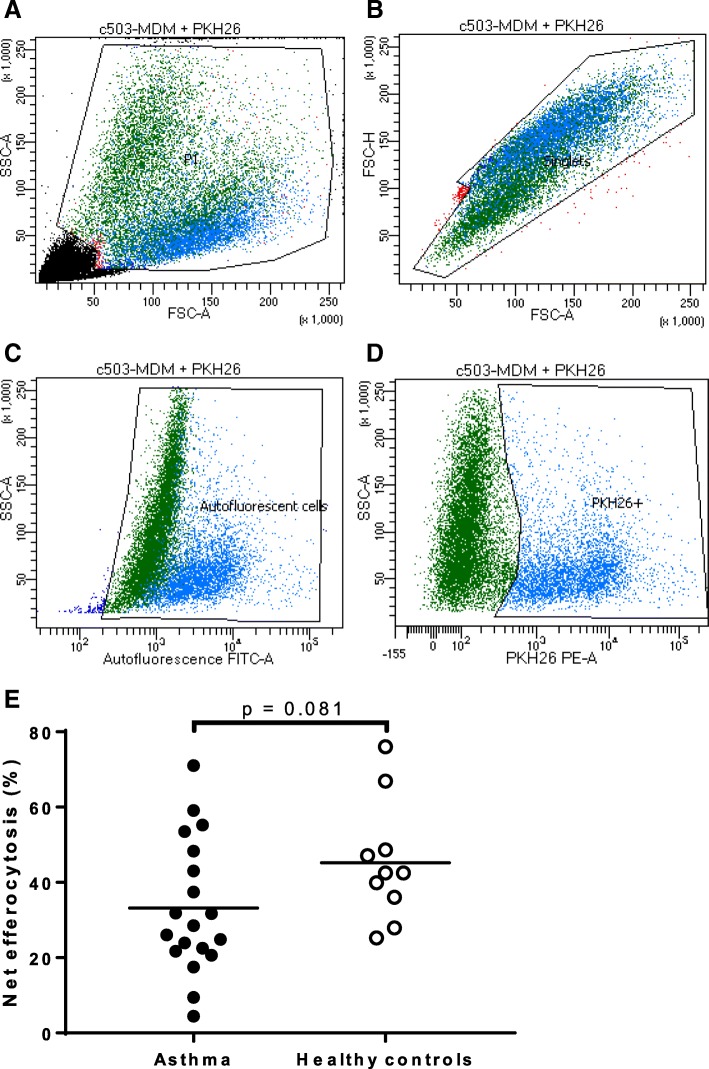
MDM efferocytosis of apoptotic granulocytes with flow cytometric gating strategy. Efferocytosis was measured in human MDMs isolated from participants with asthma (*n* = 19) and healthy controls (*n* = 10). Human MDMs were co-cultured with PKH-26-labelled apoptotic granulocytes for 90 min at 37 °C in a 24-well plate. The MDMs were harvested and efferocytosis measured by flow cytometry. (**a-b**) MDMs were gated based on size and granularity using FSC-A vs SSC-A to eliminate debris, doublets and unbound granulocytes. (**c**) Autofluorescent cells were sub-gated on a FITC plot and (**d**) the PKH-26 positive MDMs gated on a separate PE plot. (**e**) The percentage efferocytosis is calculated by the proportion of PKH26-positive MDMs minus the negative control with cytochalasin D. Bars show the means. FSC-A: forward scatter area; FSC-H: forward scatter height; SSC-A: side scatter area

MDM net efferocytosis was measured at baseline and was found to be lower in the asthma cohort (33.2 (±17.7)%) compared with the healthy controls (45.3 (±15.9)%) although the difference did not reach statistical significance (*p* = 0.081) (Fig. 1). Net efferocytosis MFI was used to estimate the relative number of engulfed granulocytes per MDM and was also observed to be lower in participants with asthma (55 (26, 352)) compared with the healthy cohort (204 (150, 488)) but did not reach statistical significance (*p* = 0.064). Net efferocytosis was not associated with any of the clinical outcomes measured such as age, BMI, lung function, ACQ6 score and ICS dose [see Additional file 1].

When examined by asthma phenotype, net efferocytosis did not differ between the EA (31.4 (±19.2)%) and NEA phenotypes (28.7 (±21.5)%; *p* = 0.748). Net efferocytosis was similar between the NEA (28.7 (±21.5)%) and healthy controls (45.3 (±15.9)%; *p* = 0.105). The net efferocytosis MFI was also similar between the EA (78 (32, 352)) and NEA phenotypes (55 (26, 832); *p* = 0.809). Net efferocytosis MFI was lower in NEA (55 (26, 832)) compared with the healthy controls (204 (150, 488)) but did not reach statistical significance (*p* = 0.088).

### Galectin-3 enhances MDM efferocytosis in asthma

**Fig. 2:**
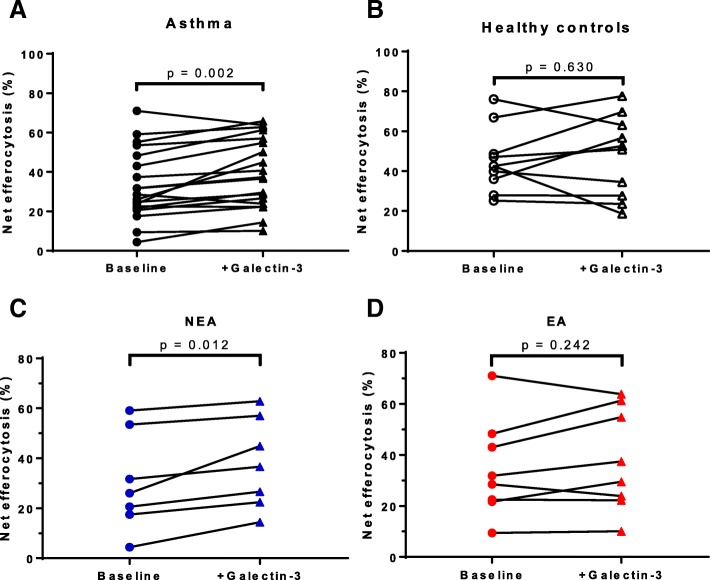
Effect of galectin-3 on MDM efferocytosis in asthma. MDMs from participants with (**a**) asthma (*n* = 19) and (**b**) healthy controls (*n* = 10), were pre-incubated with 50 μg/mL recombinant galectin-3 for 10 min at 37 °C and co-cultured with PKH-26-labelled apoptotic granulocytes for 90 min at 37 °C. The MDMs were harvested and efferocytosis measured by flow cytometry. MDMs with surface bound but non-internalised granulocytes were excluded using cytochalasin D negative control. The asthma group was split into (**c**) NEA (*n* = 7) and (**d**) EA (*n* = 8) and efferocytosis examined. Galectin-3 significantly increased efferocytosis in asthma, particularly NEA but not in the healthy controls

A concentration of 50 μg/mL galectin-3 used in this study was similar to levels reported at inflammatory sites [11, 22]. Addition of exogenous galectin-3 significantly improved net efferocytosis (39.6 (±17.8)%) compared with untreated MDMs (baseline: 33.2 (±17.7)%; *p* = 0.002) in the cohort with asthma (Fig. 2a). This represented a 19% increase from baseline. Galectin-3 had no effect on MDM net efferocytosis (47.5 (±20.4)%) compared with untreated cells (baseline: 45.3 (±15.9)%; *p* = 0.630) in the healthy controls (Fig. 2b). Net efferocytosis MFI was more than fourfold higher upon addition of galectin-3 (227 (46, 918)) which was statistically significant compared with untreated MDMs (baseline: 55 (26, 352); *p* < 0.001) in the asthma cohort. Net efferocytosis MFI was twofold higher in the galectin-3-treated group (526 (39, 1098)) compared with the untreated MDMs (baseline: 205 (150, 488)) in the healthy cohort but did not reach statistical significance (*p* = 0.275).

Galectin-3 significantly improved net efferocytosis (37.8 (±18.1)%) compared with untreated MDMs (baseline: 30.4 (±19.7)%; *p* = 0.012) in participants with NEA (Fig. 2c). This represented a 24% increase in efferocytosis from baseline. No significant improvement in net efferocytosis was observed in the galectin-3-treated group (37.9 (±20.0)%) compared with untreated MDMs (baseline: 34.5 (±19.2)%; *p* = 0.242) in participants with EA (Fig. 2d).

Addition of galectin-3 increased net efferocytosis MFI three-fold (151 (32, 660)) compared with the untreated MDMs (baseline: 47 (26, 764); *p* = 0.078) in the NEA cohort. In the EA cohort, galectin-3 treatment also improved net efferocytosis MFI three fold (291 (47, 928)) compared with the untreated MDM group (baseline: 75 (28, 314)) but did not reach statistical significance (*p* = 0.078).

**Fig. 3:**
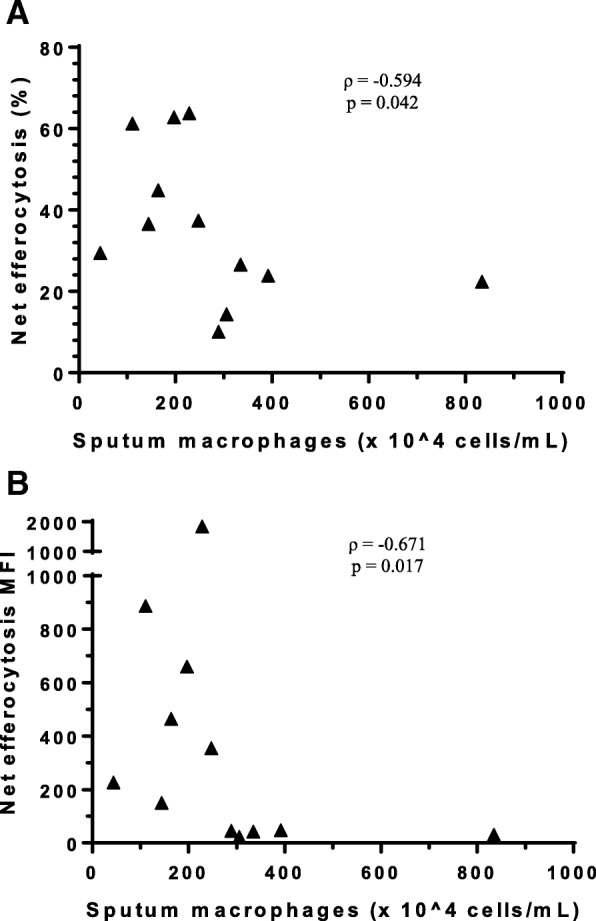
Association between net efferocytosis and sputum macrophages in asthma. The number of sputum macrophages in participants with asthma (*n* = 12) was negatively associated with (**a**) net efferocytosis % and (**b**) net efferocytosis MFI. Net efferocytosis was calculated as the difference in efferocytosis between the total efferocytosis and the cytochalasin D negative control

Net efferocytosis was not associated with any of the clinical outcomes measured. However, both net efferocytosis % and MFI following galectin-3 addition were negatively correlated with sputum macrophage numbers in participants with asthma (Spearman *r* = − 0.594; *p* = 0.042 and Spearman *r* = − 0.671; *p* = 0.017 respectively) (Fig. 3) [see Additional file 2].

### Galectin-3 expression in MDMs

**Fig. 4:**
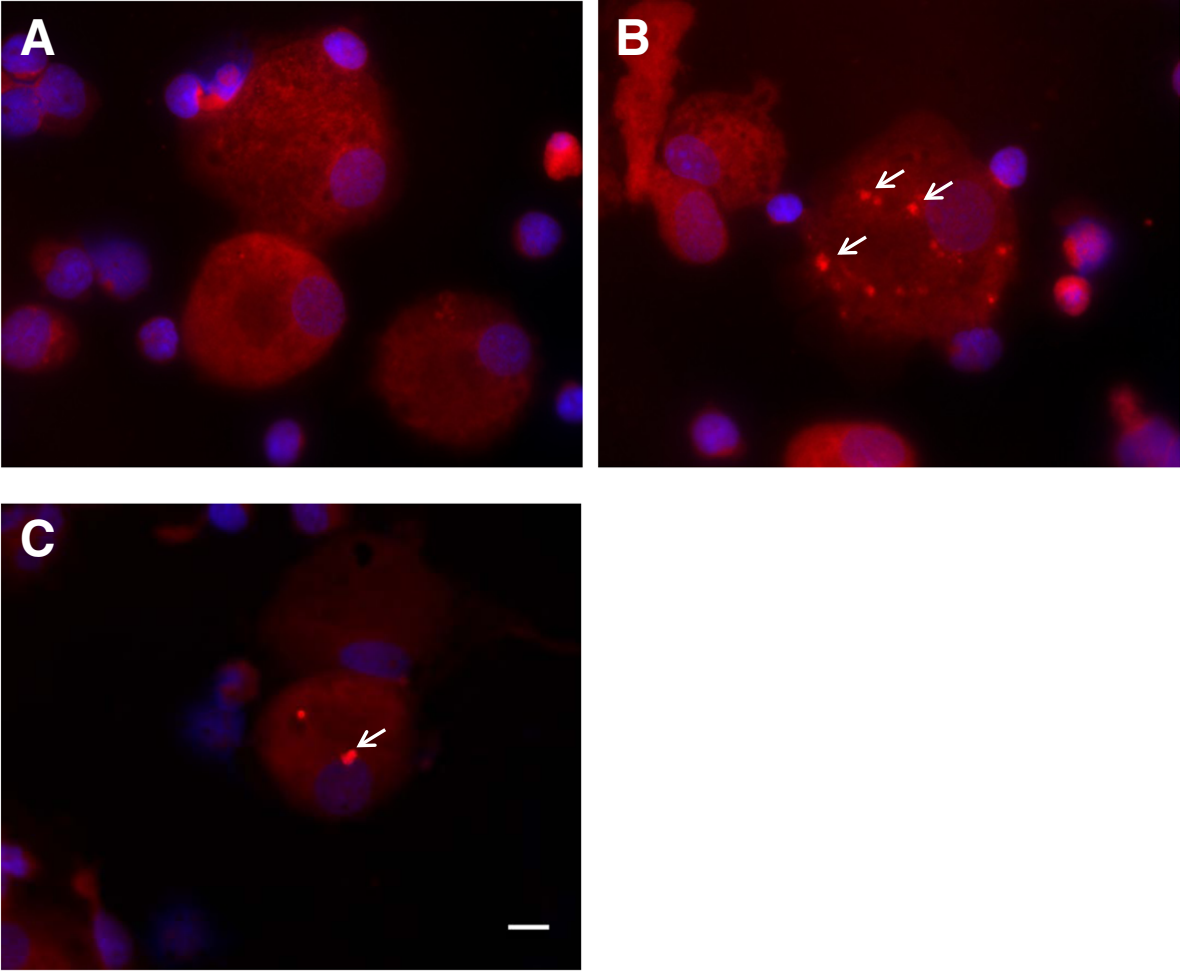
Galectin-3 expression in MDMs. MDMs were obtained from blood taken from participants with asthma (*n* = 18) and healthy controls (*n* = 6). MDMs were transferred to cytospin slides, fixed and permeabilised with PLP. The cells were immunostained with a PE conjugated rat anti-human galectin-3 antibody (2.5 μg/mL) shown in red and the nuclei stained with DAPI in blue. (**a-b**) MDMs from the same healthy participant showing galectin-3 puncta (white arrows). (**c**) MDMs stained with a galectin-3-PE antibody from a different manufacturer also showing punctae. Scale bar, 10 μm (original magnification × 1000)

Galectin-3 had a diffuse but uniform distribution in the cytoplasm and nucleus of most MDMs, however some cells appeared to have concentrated areas of galectin-3 staining visible under high magnification (× 1000) (Fig. 4a-b). A similar pattern was observed in MDMs when stained with another PE-conjugated galectin-3 antibody from a different manufacturer, confirming that the galectin-3 puncta were not an artefact of the staining process (Fig. 4c). A corresponding PE isotype antibody was also used to ascertain that the galectin-3 staining observed was specific and showed negligible staining in MDMs. The proportion of MDMs with galectin-3 punctae was similar between the asthma (4.8 (0.9, 6.7)%) and healthy cohorts (4.8 (0.2, 7.3)%; *p* = 0.845). The proportion of punctate MDMs was not associated with any of the clinical outcomes, efferocytosis or sputum cell counts.

**Fig. 5:**
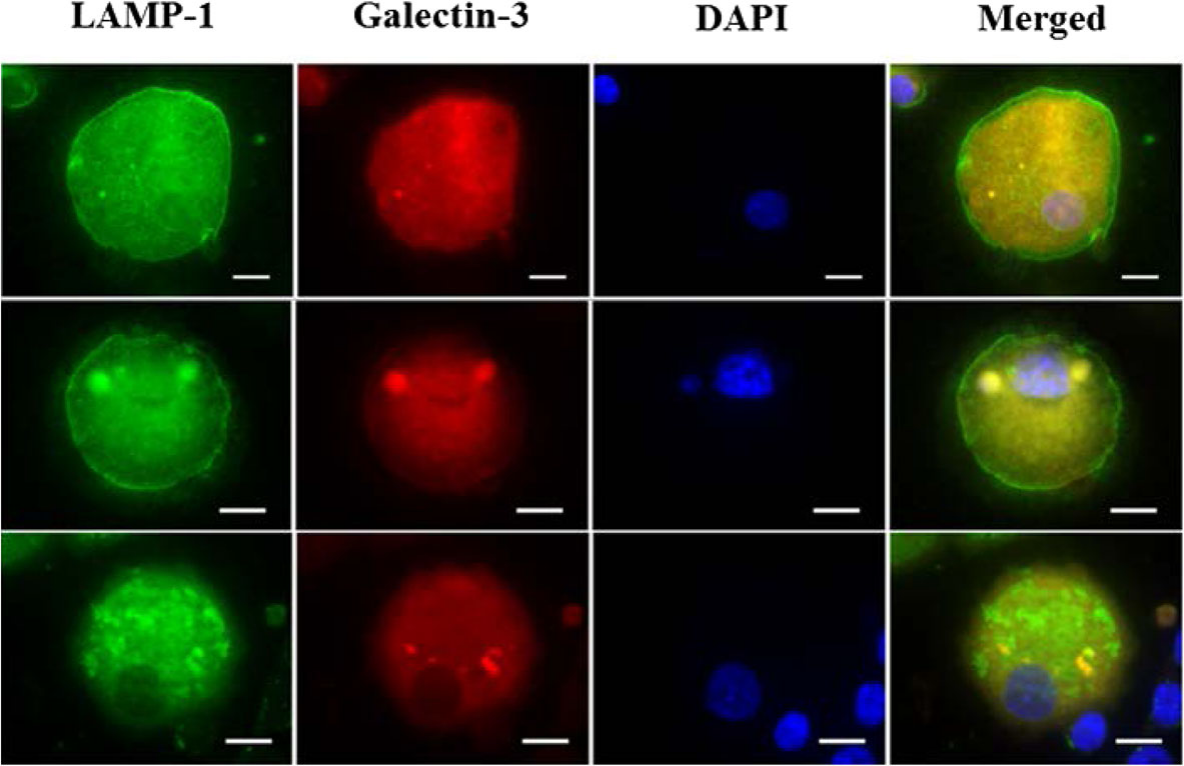
Expression of galectin-3 and LAMP-1 in permeabilised MDMs. Double immunofluorescence staining of MDMs with LAMP-1 (green), galectin-3 (red) and DAPI nuclear staining (blue). Co-localisation of galectin-3 and LAMP-1 was investigated by fluorescent microscopy and was identified as yellow punctate staining in the cytoplasm of MDMs. Scale bar, 10 μm

Galectin-3 has previously been reported to be associated with intracellular compartments such as phagolysosomes in macrophages [8]. In order to determine the localisation of the galectin-3 puncta in MDMs, a galectin-3/LAMP-1 double indirect immunofluorescence staining was performed. LAMP-1 is an intracellular marker found on the lumenal side of lysosomes [23]. Galectin-3 and LAMP-1 were found to be expressed in all MDMs and the punctate structures generally appeared yellow due to the superimposition of the red and green colour (Fig. 5).

### Inflammatory mediator secretion by MDM

**Fig. 6:**
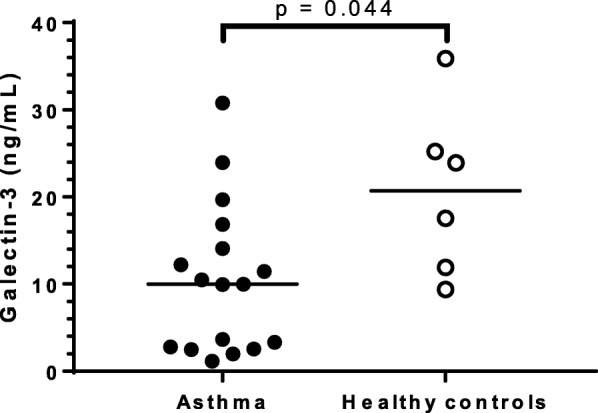
Galectin-3 secretion by MDM in asthma MDM cell culture supernatants from participants with asthma (*n* = 17) and healthy controls (*n* = 6) were collected on Day 12 prior to the efferocytosis assay and galectin-3 levels were measured by ELISA. Galectin-3 secretion was significantly lower in asthma. Bars show the medians

Galectin-3 levels were measured in MDM supernatant 12 days post cell isolation (and following 3–4 days of culture) prior to efferocytosis and were found to be significantly lower in asthma (9.99 (2.67, 15.48) ng/mL) compared with healthy controls (20.72 (11.28, 27.89) ng/mL; *p* = 0.044) (Fig. 6). There was no significant difference in MDM galectin-3 levels between the EA (3.63 (2.48, 14.09) ng/mL) and NEA participants (9.96 (2.56, 16.86) ng/mL; *p* = 1.000). About 40% of the participants with asthma secreted very little galectin-3 compared with the rest of the participants but were comprised of a mix of both EA and NEA participants. Galectin-3 secretion was positively associated with the number of MDMs per well (Spearman *r* = 0.533; *p* = 0.011) but was not associated with efferocytosis. The number of MDMs per well did not differ between the asthma (2.3 × 10^5^ (±1.1 × 10^5^) cells/well) and healthy controls (2.0 × 10^5^ (±1.2 × 10^5^) cells/well; *p* = 0.612). To account for the differences in MDM numbers per well, galectin-3 secretion was recalculated as the amount secreted per 10^5^ cells and was still observed to be significantly lower in asthma (3.38 (1.74, 6.69) ng/mL/10^5^ MDMs) compared with the healthy controls (11.06 (4.9, 14.06) ng/mL/10^5^ MDMs; *p* = 0.031). There was no significant difference in galectin-3 secretion per 10^5^ MDMs between the EA and NEA phenotypes.

MDM secreted IL-6 and CXCL8 levels were similar between the asthma and healthy participants [IL-6 (asthma: 59.8 (27.6, 92.9) pg/mL and healthy participants: 45.2 (30.6, 569.9) pg/mL; *p* = 1.000) and CXCL8 (asthma: 4.37 (1.73, 10.44) ng/mL and healthy participants: 7.57 (1.88, 15.40) ng/L; *p* = 0.703)]. There were no statistically significant differences in IL-6 and CXCL8 levels between the asthma and healthy controls when recalculated per 10^5^ MDMs. IL-6 and CXCL8 were not associated with efferocytosis (Spearman *r* = 0.113; *p* = 0.626 and Spearman *r* = 0.404; *p* = 0.107 respectively). IL-1β, IL-10, IL-17A, IFN-ɣ and TNF were undetectable in MDM supernatant.

## Discussion

Previous studies have shown that AM efferocytosis is reduced in asthma, particularly non-eosinophilic asthma [4]. In this study, we observed reduced MDM efferocytosis from participants with asthma and demonstrated that addition of galectin-3 could significantly improve efferocytosis in asthma but not in healthy MDMs. This finding suggests that efferocytosis may already be occurring at the maximal rate in healthy MDMs and cannot be further increased, or at least not to a detectable level using this assay. Galectin-3 has previously been reported to enhance MDM efferocytosis of apoptotic granulocytes in a concentration-dependent manner [11]. In this study upon galectin-3 addition, MDM efferocytosis in participants with asthma was increased by almost 20% of baseline but did not reach baseline efferocytosis measured in the healthy controls. This provides further evidence of a macrophage defect observable at the systemic level in asthma [4, 21]. The cohort with asthma were mostly GINA 4 with persistent symptoms and more severe disease, which may influence inflammatory cell regulation, exemplified by a decrease in macrophage efferocytosis. Regulating macrophage function may be the key to regulating inflammation and the associated symptoms in asthma.

In NEA, defective efferocytosis may lead to an accumulation of dead cells in the airways which may undergo secondary necrosis and release pro-inflammatory mediators and damage-associated molecular patterns [5, 24]. These molecules activate and recruit more inflammatory cells to the lungs that may in turn lead to persistent airway inflammation. In this study, there was no link between MDM efferocytosis and MDM IL-6 and CXCL8 secretion, while the remaining cytokines (IL-1β, IL-10, IL-17A, IFN-ɣ and TNF) were undetectable in MDM supernatant. However, a negative correlation between efferocytosis and sputum macrophage numbers was observed. This finding may suggest an increased recruitment, proliferation or survival of macrophages in the airways in response to decreased efferocytosis [25]. A similar effect has been reported in COPD, whereby lung AM numbers were linked to the severity of the condition [26]. It is possible that defective efferocytosis may lead to the generation of chemotactic or proliferative signals that promote macrophage migration to the airways.

Galectin-3 has been reported to accumulate at different sites within cells such as phagosomes [8], damaged vesicles [27] and pathogen-containing vacuoles [27, 28]. In this study, galectin-3 was diffusely distributed in the cytoplasm and nucleus of most MDMs which is in agreement with the localisation previously reported in macrophages [8, 29, 30]. Galectin-3 is known to shuttle between the nucleus and cytoplasm depending on the cell type and culture conditions [31]. In this study, approximately 5% of MDMs also exhibited galectin-3 punctae which did not differ between the groups, indicating that galectin-3 trafficking may be occurring at a normal rate in asthma. The proportion of MDMs with punctae was not associated with efferocytosis which makes it difficult to predict the effect of galectin-3 punctae on this process, if any. The galectin-3 punctae also tended to co-localise with LAMP-1 in MDMs which agrees with previous studies that suggest that it may be localised to phagolysosomes [8]. Further investigations using confocal microscopy might help pinpoint the exact location of galectin-3 punctae in macrophages and its dynamic movement within the cell.

Reduced levels of galectin-3 in sputum have been reported in asthma and are thought to contribute to persistence of airway inflammation through decreased macrophage efferocytosis [4, 12]. In this study, galectin-3 secretion by MDMs was lower in participants with asthma compared with healthy participants, while IL-6 and CXCL8 secretion was similar between the groups. These findings suggest that although galectin-3 trafficking in MDMs from participants with asthma is occurring at rate similar to healthy controls, the amount secreted is significantly less which may affect the function of the cells exemplified in this case as decreased efferocytosis in asthma.

A limitation of this study is the high BMI of the participants with asthma which may have an effect on immunological processes on the body. In fact, a higher BMI is generally associated with a more pro-inflammatory immune response such as increased TNF-α and IL-6 expression [32–34], which may influence galectin-3 expression in macrophages [35]. Future studies could be expanded to a larger cohort of BMI-matched participants to ensure the differences observed are driven purely by the disease state.

## Conclusions

In conclusion, this study reported no difference between MDM efferocytosis of apoptotic granulocytes in adults with asthma and healthy controls but showed that it can be increased in asthma, particularly in NEA through the addition of exogenous galectin-3. These findings indicate that galectin-3 may play a role in macrophage efferocytosis and may provide an indirect way of modulating inflammation in asthma.

## Additional files

Additional file 1: Correlations between net efferocytosis and clinical outcomes in participants with asthma. (DOCX 15 kb)

Additional file 2: Correlations between net efferocytosis and sputum cell numbers in participants with asthma. (DOCX 14 kb)

## Abbreviations

ACQ6: Asthma control questionnaire 6
AM: Airway macrophage
BMI: Body mass index
CO: Carbon monoxide
COPD: Chronic obstructive pulmonary disease
DAPI: 4′,6-diamidino-2-phenylindole
EA: Eosinophilic asthma
ELISA: Enzyme-linked immunosorbant assay
FBS: Foetal bovine serum
FVC: Forced vital capacity
GINA: Global initiative for asthma
GM-CSF: Granulocyte macrophage-colony stimulating factor
ICS: Inhaled corticosteroid
IL: Interleukin
LAMP-1: Lysosomal-associated membrane protein 1
MDM: Monocyte-derived macrophages
MFI: Median fluorescence intensity
N/A: Not applicable
NEA: Non-eosinophilic asthma
PBMC: Peripheral blood mononuclear cell
PBS: Phosphate buffered saline
PE: Phycoerythrin
ppm: parts per million
RPMI: Roswell Park Memorial Institute
SD: Standard deviation
