# Supplementary material for: Galectin-3 enhances monocyte-derived macrophage efferocytosis of apoptotic granulocytes in asthma
Source: Respir Res. 2019 Jan 3;20:1. doi: 10.1186/s12931-018-0967-9 (PMC6318889; doi:10.1186/s12931-018-0967-9)
Supplement: Supplementary file 1 — Correlations between net efferocytosis and clinical outcomes in participants with asthma. (DOCX 15 kb) [file 12931_2018_967_MOESM1_ESM.docx]

**Correlations between net efferocytosis and clinical outcomes in participants with asthma**

|  | **Net efferocytosis without galectin-3 (%)** | **Net efferocytosis MFI without galectin-3** | **Net efferocytosis**  **with galectin-3 (%)** | **Net efferocytosis MFI with galectin-3** |
| --- | --- | --- | --- | --- |
| Age, years | 0.12 | 0.09 | -0.04 | -0.11 |
| BMI | 0.01 | -0.06 | -0.11 | -0.07 |
| FEV_1_% predicted | 0.05 | 0.09 | 0.16 | 0.07 |
| FEV_1_/FVC% | 0.14 | 0.23 | 0.07 | -0.02 |
| Pack years | -0.40 | -0.21 | -0.40 | -0.40 |
| Exhaled CO, ppm | 0.40 | 0.35 | 0.45 | 0.65** |
| ICS dose, µg/day | -0.25 | -0.28 | -0.28 | -0.28 |
| ACQ score | 0.36 | 0.40 | 0.09 | 0.17 |

Spearman r values given (n = 19). BMI: body mass index; CO: carbon monoxide; FEV_1_: forced expiratory volume in one second; FVC: forced vital capacity; ICS: inhaled corticosteroid; ACQ: asthma control questionnaire. *p < 0.05, **p < 0.01.
