# Supplementary material for: Galectin-3 enhances monocyte-derived macrophage efferocytosis of apoptotic granulocytes in asthma
Source: Respir Res. 2019 Jan 3;20:1. doi: 10.1186/s12931-018-0967-9 (PMC6318889; doi:10.1186/s12931-018-0967-9)
Supplement: Supplementary file 2 — Correlations between net efferocytosis and sputum cell numbers in participants with asthma. (DOCX 14 kb) [file 12931_2018_967_MOESM2_ESM.docx]

**Correlations between net efferocytosis and sputum cell numbers in participants with asthma**

|  | **Net efferocytosis without galectin-3 (%)** | **Net efferocytosis MFI without galectin-3** | **Net efferocytosis**  **with galectin-3 (%)** | **Net efferocytosis MFI with galectin-3** |
| --- | --- | --- | --- | --- |
| Total cell count (10^6^/mL) | -0.22 | -0.14 | -0.43 | -0.48 |
| Viability (%) | -0.32 | -0.22 | -0.40 | -0.31 |
| Neutrophils (%) | -0.01 | -0.04 | -0.01 | -0.03 |
| Neutrophils (10^4^/mL) | -0.02 | 0.02 | -0.18 | -0.27 |
| Eosinophils (%) | 0.18 | 0.09 | 0.03 | 0.12 |
| Eosinophils (10^4^/mL) | 0.28 | 0.23 | 0.06 | 0.26 |
| Macrophages (%) | -0.10 | -0.01 | -0.05 | -0.09 |
| Macrophages (10^4^/mL) | -0.46 | -0.27 | -0.59* | -0.67* |
| Lymphocytes (%) | -0.21 | -0.37 | -0.19 | -0.13 |
| Lymphocytes (10^4^/mL) | -0.16 | -0.29 | -0.09 | -0.04 |
| Columnar epithelial cells (%) | -0.02 | -0.10 | 0.00 | -0.05 |
| Columnar epithelial cells (10^4^/mL) | -0.40 | -0.50 | -0.32 | -0.47 |
| Squamous cells (%) | -0.03 | -0.01 | 0.21 | 0.13 |

Spearman r values given (n = 15). *p < 0.05, **p < 0.01.
